# Marriage, contraception, and fertility decline in Egypt: sociological insights from Bongaarts’s proximate determinants framework

**DOI:** 10.3389/fsoc.2026.1883668

**Published:** 2026-09-14

**Authors:** AbdelMajeed Ahmed Hendy, Mastur Hammad Rehim, Ahmed Mohi Khalaf Sakr, Khalil Ibrahim Al-Halalat, Ruqayya Alteneiji

**Affiliations:** 1 Department of Sociology, College of Arts, Humanities, and Social Sciences, University of Al Dhaid, Sharjah, United Arab Emirates; 2 Department of Population Studies, Faculty of Arts and Humanities, Minia University, Minya, Egypt; 3 Department of Sociology, Umm Al Quwain University, Umm Al Quwain, United Arab Emirates; 4 Department of Sociology, Faculty of Arts, Minia University, Minya, Egypt; 5 Department of Social Work, The University of Jordan, Amman, Jordan; 6 College of Arts, Humanities, and Social Sciences, University of Al Dhaid, Al Dhaid, Sharjah, United Arab Emirates

**Keywords:** Bongaarts, contraception, fertility, infecundability, marriage

## Abstract

**Background:**

Egypt’s total fertility rate (TFR) increased from 3.0 births per woman in 2008 to 3.5 in 2014, before declining to 2.85 in 2021. This study examined the proximate determinants associated with the post-2014 fertility decline.

**Methods:**

The analysis used published aggregate indicators from the nationally representative EDHS 2014 and EFHS 2021, covering 21,762 and 20,481 ever-married women aged 15–49 years, respectively. Bongaarts’s proximate determinants framework was applied to marriage, contraception, postpartum infecundability, and induced abortion. Relative contributions were assessed using logarithmic decomposition, and sensitivity analysis evaluated total fecundity (TF) values of 15.3 and 17.

**Results:**

The marriage index declined from 0.56 to 0.39, contraceptive prevalence increased from 58 to 66%, and the contraceptive index declined from 0.41 to 0.34. Weighted contraceptive effectiveness decreased slightly from 0.935 to 0.921, while the postpartum infecundability index declined from 0.79 to 0.77. Marriage accounted for 63.7% of the model-implied fertility change, contraception 32.2%, and postpartum infecundability 4.1%. However, the model-estimated TFR declined from 2.82 to 1.58, indicating that the measured determinants did not fully reproduce the observed fertility trajectory.

**Conclusion:**

Egypt’s post-2014 fertility decline was associated mainly with reduced marriage exposure and stronger contraceptive inhibition, while postpartum infecundability played a limited role.

## 1 Introduction

Worldwide fertility rates have decreased, but the speed and reasons differ across demographic, socioeconomic, and reproductive settings (Rehim et al., 2025). Egypt stands out as a significant example due to fertility stagnating prior to another decline, while swift population growth continues to be a key developmental issue. The TFR decreased to 3.0 births per woman in 2008, increased to 3.5 in 2014, and dropped to 2.85 in 2021 (Abdel-Tawab et al., 2020; Central Agency for Public Mobilization and Statistics (CAPMAS), 2022; Ministry of Health and Population [Egypt] et al., 2015). As of 2024, Egypt’s population hit around 107 million, with population growth continuing to be a key policy focus, as seen in the Population and Development Plan initiated in 2025 (Ahram Online, 2024; Central Agency for Public Mobilization and Statistics (CAPMAS), 2024; Top 50 Women Forum, 2025). Fertility is influenced through a distinction of background factors such as socio-economic status, level of education, reproductive desire and access to healthcare services versus the direct influence of the four key proximal factors on exposure to conception. Marriage/union exposure, use of contraception, post-partum infertility and induced abortion are identified in the Bongaarts framework as being the primary proximal factors which decrease observed fertility compared with the estimated total fecundity (TF) for women at approximately 15.3 births (Bongaarts and Potter, 1983).

The framework was selected for the present study because it provides a standardized quantitative approach for distinguishing and comparing the direct demographic mechanisms through which fertility is inhibited. In particular, it permits changes in marriage exposure, contraception, postpartum infecundability, and induced abortion to be evaluated within a common multiplicative structure using indicators available from nationally representative fertility surveys. This makes the framework especially suitable for examining which proximate mechanisms were associated with Egypt’s renewed fertility decline between 2014 and 2021, while recognizing that broader socioeconomic and sociocultural determinants operate through these proximate pathways.

The main focus of Egypt’s family planning programs have been on methods of birth control. Therefore, IUDs, oral contraceptives and injectable contraceptives are commonly used throughout the country. However, since a woman’s method of choice and whether or not she continues to use her chosen form of contraception can be influenced by factors such as education level, place of residency, social class, ability to access services and possible negative side effects of the chosen form of contraception in addition to reproductive preferences, it is important that both contraceptive prevalence rates and the effectiveness/continuation of chosen forms of contraception are taken into consideration when assessing the impact that they have had on reducing overall fertility in Egypt (Ministry of Health and Population [Egypt] et al. 2015; Central Agency for Public Mobilization and Statistics (CAPMAS), 2022).

An excellent opportunity exists for studying Egypt’s renewed reduction in fertility following a prolonged plateau beginning with the year 2014 through 2021. Prior studies applied the Bongaarts framework to prior stages of Egypt’s fertility transition; however, none included data collected in the EFHS 2021 survey. Accordingly, an empirical gap remains in understanding the proximate demographic mechanisms associated specifically with Egypt’s post-2014 fertility decline. In particular, the relative changes in marriage exposure, contraceptive use and method effectiveness, postpartum infecundability, and induced abortion have not previously been examined together using EFHS 2021 indicators within the Bongaarts framework. This study represents an extension of prior work examining the proximate determinants of fertility decline by applying the same framework to nationally representative data collected via the EDHS 2014 and EFHS 2021 surveys to explain the behavioral pathways contributing to fertility decline in the post-stall phase.

The study period also coincided with an increased national focus on population policy, including the “Two Children Only” campaign initiated in 2017, investments aimed at improving citizens’ quality of life and economic conditions, and the effects of COVID-19 (Arab News, 2017; Egypt Today, 2022; Dyer, 2023). Although this study cannot determine whether these contextual events caused the observed changes in marriage patterns, contraceptive behavior, and fertility levels, they provide an important background for interpreting trends between 2014 and 2021. There were two goals/objectives for this study: First, to measure changes in the four proximal determinant indices--i.e. marriage/exposure to union, contraception and effectiveness, postpartum infecundability and induced abortion -- between 2014 and 2021. Second, to break down the change in total fertility observed during these years and evaluate what behavioral pathways contributed most significantly to Egypt’s post-stall decline in fertility.

## 2 Literature review

### 2.1 Theoretical foundations and critiques

The proximate determinants framework, originally developed by Davis and Blake (1956) as “intermediate variables,” was refined by Bongaarts (1978, 1982) into a quantitative model for analyzing fertility variations across populations (International Union for the Scientific Study of Population (IUSSP), n.d.). Bongaarts (1978) identified seven proximate determinants of fertility: percentage of women who are married, method and efficacy of contraception, postpartum period of infertility, abortion, fecundability, sterility, and intrauterine mortality. Among these, four determinants—marriage, contraception, postpartum infecundability, and induced abortion—are considered most important, accounting for approximately 96 percent of fertility variations across populations (Bongaarts, 1982; Weeks, 2001).

Despite its widespread use, the proximate determinants framework has faced several critiques. First, the model assumes that fertility-inhibiting behaviors are exogenous to fertility preferences. However, recent research suggests that fertility preferences and the implementation of those preferences are causally related and endogenous (Bongaarts, 2024). As Bongaarts (2024) argues, “declining wanted fertility appears to induce a contraceptive response,” meaning that demand for children and contraceptive implementation cannot be treated as independent factors. Studies that assume independence between demand and implementation likely underestimate the role of preferences (N-IUSSP, 2024).

Second, the proximate determinants model has recognized limitations in certain contexts. For example, high levels of primary and secondary sterility in parts of Africa may elevate the importance of sterility as a proximate determinant beyond the four standard variables (Blank & Poukouta, 1997). Additionally, sexual activities are often not confined only within marriage, meaning the marriage index may not fully capture exposure to sexual intercourse (International Union for the Scientific Study of Population (IUSSP), n.d.).

Alternative frameworks have been developed to address these limitations. Easterlin’s “Synthesis Framework” (Easterlin, 1983) is an effort to integrate proximate determinants into a larger socioeconomic context, where child demand, child supply, and contraception cost are related to actual levels of fertility (Bongaarts, 2024). The intermediate variables framework developed by Davis and Blake provided the conceptual foundation for Bongaarts’ later quantitative formulation (Weeks, 2001; Bongaarts, 1987). The proximate determinants framework continues to be widely used in decomposition analysis because of its practical applicability and clear analytical structure. First, as Bongaarts (2024) notes, longitudinal studies of trends in wanted and unwanted fertility are methodologically less problematic than cross-sectional analyses. Second, the framework’s focus on four key variables allows for relatively simple decomposition using widely available survey data, making it accessible for policy-relevant research in low- and middle-income countries. Third, while alternative frameworks offer richer theoretical accounts of socioeconomic determinants, the proximate determinants approach provides clear quantification of the behavioral channels through which fertility change occurs.

### 2.2 Empirical applications

Several studies have applied the proximate determinants framework to Egypt. Zaky (1999) used Bongaarts’s indices to examine fertility change from 1980 to 1995 and found that contraception was the main factor behind the decline in fertility, even though its impact diminished as time went on. Marriage exerted a notable and geographically diverse impact on fertility rates.

Eltigani (2003), utilizing DHS data from Egypt (1988, 1992, 1995 and 2000) focused on explaining the “stalled” decline in total fertility rates observed in Egypt since the mid-1990s. El Tigani’s research concluded that reproductive behaviors exhibited by women residing in higher and middle standards of living household were primarily responsible for the stall in fertility decline.

Eltigani (2009) reported that the difference in the pace of fertility decline between Tunisia and Egypt was partly explained by the greater increase in women’s age at first marriage in Tunisia. The study concluded that achieving replacement-level fertility in Egypt would depend on further reductions in total marital fertility through lower desired fertility and improved access to family planning services. Rashad and Eltigani (2005) also examined the relationship between population policy, cultural dynamics, and fertility decline in Egypt.

The most recent proximate determinant’s (PD) analysis of Egypt’s fertility trend since 2000 concluded that changes in contraceptive use were the largest and increasing suppressive factor affecting fertility prior to 2000, however; there has been little if any change in the relationship between marriage and/or contraceptive use and fertility from 2000 onward. The study attributed the fertility stall to well-educated women aged 20–29 lacking labor market opportunities, potentially leading them to prepone their fertility.

The present study extends this line of research by examining the 2014–2021 period, during which Egypt experienced renewed fertility decline after the post-2000 stall, and by applying the Bongaarts framework to the most recent Egyptian Family Health Survey data that have not previously been analyzed using proximate determinants decomposition.

Cross-national applications exhibit significant differences in the primary proximate factor. Rogers and Stephenson (2018) recorded changes over time in postpartum factors across 33 low- and middle-income nations. In Ethiopia, marriage was significant in 2011, whereas postpartum infecundability prevailed in 2016 (Laelago et al., 2019); marriage also held significance in Namibia and Timor-Leste (Palamuleni, 2017; Hogan et al., 2025). In Eswatini and Mongolia, contraception was prevalent (Chemhaka and Odimegwu, 2019; Spoorenberg, 2009), while in Peninsular Malaysia and Malawi, both marriage postponement and contraception played significant roles (Tey et al., 2012; Palamuleni, 2010). Applications to South Korea and the United States also demonstrate that the framework is not limited to high-fertility contexts (Guarneri, 2010) (see Table 1).

**Table 1:** Summary of cross-national applications of the Bongaarts proximate determinants framework.

Country	Primary inhibitor	Citation
Ethiopia	Marriage (2011), Postpartum (2016)	Laelago et al. (2019)
Eswatini	Contraception	Chemhaka and Odimegwu (2019)
Namibia	Marriage	Palamuleni (2017)
Timor-Leste	Marriage	Hogan et al. (2025)
Mongolia	Contraception	Spoorenberg (2009)

### 2.3 Methodological debates

The Bongaarts proximate determinants framework has been subject to ongoing methodological debate, particularly regarding two key assumptions: the value of total fecundity (TF) and the estimation of contraceptive effectiveness.

### 2.4 The Total fecundity (TF) assumption

According to Bongaarts and Potter (1983), total fecundity (TF) represents the fertility level that would prevail in the absence of the fertility-inhibiting effects represented by the principal proximate determinants. In the original aggregate model, TF is conventionally set at 15.3 births per woman. TF must be distinguished from the total fertility rate (TFR): TFR is the observed period measure of births per woman under prevailing fertility conditions, whereas TF is a counterfactual model parameter representing fertility in the absence of the modeled inhibiting effects. The value of 15.3 was derived from empirical evidence on populations characterized by natural fertility and became the conventional standard used in applications of the Bongaarts framework (Bongaarts and Potter, 1983).

Alternative values for total fecundity (TF) were suggested for certain cases by others. The author of a study on fertility inhibiting indexes in 16 Arab countries; that included Egypt, Horne, (1993) used a total fecundity value of 17 rather than the standard TF value of 15.3 as recommended by Bongaarts in the application to Arab populations. The value of 17 should not, however, be interpreted as an independently established biological maximum that is necessarily more appropriate for Egypt. Rather, it represents an alternative calibration toward the upper end of the range of total fecundity values considered plausible in applications of the model. Avoidance of negative estimated percentage reductions alone is insufficient grounds for treating 17 as the biologically correct value. Accordingly, the difference between TF = 15.3 and TF = 17 is treated in the present study as a model-specification issue rather than as a distinction between an incorrect and a correct biological maximum.

Bongaarts returned to his own model in 2015 with six modifications to improve the proximate determinants framework based on new research and changes in sexual behavior over the last 30 years (Bongaarts, 2015). The modified version was then used by Bongaarts to provide the latest available estimates of the indexes of proximate determinants and total fertility for 36 developing countries, all of which were drawn from recent DHS surveys. Bongaarts also said that while “the original Proximate Determinants Model remains conceptually correct,” there is both theoretical and empirical support for modifying the model to increase its accuracy and strength within modern population groups (Bongaarts, 2015).

For Egypt specifically, the appropriateness of the TF = 15.3 assumption deserves consideration. The decline in Egypt’s observed TFR from 5.21 in 1980 (Horne, 1993) to 2.85 in 2021 does not by itself demonstrate that the underlying TF parameter has declined, because observed TFR and model-assumed total fecundity represent conceptually different quantities. The choice of TF should therefore be evaluated as an assumption of the proximate-determinants model rather than inferred directly from Egypt’s observed fertility level. Bongaarts (2015) provides some empirical support for retaining the conventional specification: across the developing-country populations examined in the revised framework, the estimated average TF was 15.4, close to the original value of 15.3. However, following Bongaarts (2015) and the preponderance of cross-national applications, the standard TF = 15.3 assumption was retained in this analysis while acknowledging this limitation. Given Horne’s alternative specification for Arab populations, the robustness of the model estimates to TF = 17 is additionally examined through sensitivity analysis.

### 2.5 The contraceptive effectiveness adjustment

In the calculation of the contraceptive index (Cc), the following formula is used: Cc = 1 – (1.08 × U × e), where 1.08 is the factor used in calculating the index. In the initial Bongaarts aggregate model, the 1.08 multiplier serves as a fecundity correction that is applied to the multiplication of contraceptive prevalence (*U*) and average contraceptive effectiveness (*e*); it must not be viewed as a modification for inadequate contraceptive adherence. The effectiveness specific to each method is indicated by *e*, while the 1.08 multiplier is included in the model’s adjustment of the predicted fertility-inhibiting impact of contraception (Bongaarts and Potter, 1983; Bongaarts, 2015).

While this adjustment has been utilized in applications of the Bongaarts model it is time to examine whether or not the adjustments remain valid for today’s context. The mix of contraceptive methods available as well as usage patterns may have changed since the development of the adjustment. Additionally, the original derivation of the multiplier involved populations that had a vastly different method mix than current day Egypt, which includes IUDs and injectable contraceptives. Although, the 1.08 factor was maintained in the main analysis due to its inclusion within the standard aggregate Bongaarts model utilized in the research; thus allowing for comparable results across both the original and modified models. Therefore, maintaining this factor does not suggest that this factor has been independently validated for use in today’s Egyptian context. The application of a constant adjustment for fecundity will potentially introduce additional uncertainty with respect to the size of the Cc parameter estimate; as well as the resulting model-estimate of the Total Fertility Rate (TFR) based upon a given set of parameters. Overall, it is clear from studies using the Bongaarts framework across countries that there is a great deal of diversity in what proximate factor drives population-level declines in fertility. Typically, use of contraception is dominant when successful family planning programs exist, while delaying marriage is typically dominant when there is an accelerated shift to higher ages at first marriage. Postpartum infecundability also appears to have less of a role than either of these factors, which underscores why country specific decompositions as presented here are so important.

## 3 Methodology

### 3.1 Data source

#### 3.1.1 Survey descriptions

The 2014 EDHS conducted interviews with 21,762 women aged 15–49 years who have ever been married. It utilized a nationally representative, stratified, two-stage cluster sampling technique to produce estimates for Egypt, its main geographic regions, and particular governorates. Out of the 21,903 eligible women identified, 21,762 participated in the interview, yielding a response rate of 99.4% (Ministry of Health and Population [Egypt] et al., 2015).

The 2021 EFHS interviewed 20,481 ever-married women aged 15–49 years and was designed to provide nationally representative estimates for Egypt’s geographic regions. No response rate for the women’s survey was identified in the publicly available EFHS documentation; therefore, none was inferred (Central Agency for Public Mobilization and Statistics (CAPMAS), 2022).

According to the survey documentation, both surveys used nationally representative sampling designs. The present study did not analyze respondent-level microdata; instead, it used published aggregate fertility, marriage, contraceptive, and postpartum indicators from the official EDHS 2014 and EFHS 2021 reports. These published estimates already incorporated the surveys’ weighting and design procedures; therefore, no additional individual-level weighting or complex-survey adjustment was performed (Central Agency for Public Mobilization and Statistics (CAPMAS), 2022; Ministry of Health and Population [Egypt] et al., 2015).

#### 3.1.2 Data access, ethics, and missing data

Approval to conduct this study ethically was granted by the Vice Chancellor for Academic Affairs of the University of Al Dhaid (United Arab Emirates) on 13 May 2026. The approval referenced compliance with the Declaration of Helsinki and UAE National Research Ethics Regulations. The study used anonymized secondary data and involved no direct participant contact; therefore, no additional recruitment or informed consent was required. No approval reference number was provided in the approval documentation.

The analysis relied on aggregate indicators reported in published survey tables. Therefore, no individual-level exclusions or missing-data imputations were performed. When a required indicator was unavailable, the corresponding model assumption was explicitly identified. Figure 1 summarizes the data sources, extraction of aggregate indicators, and their use in the proximate determinants analysis.

**Figure 1:**
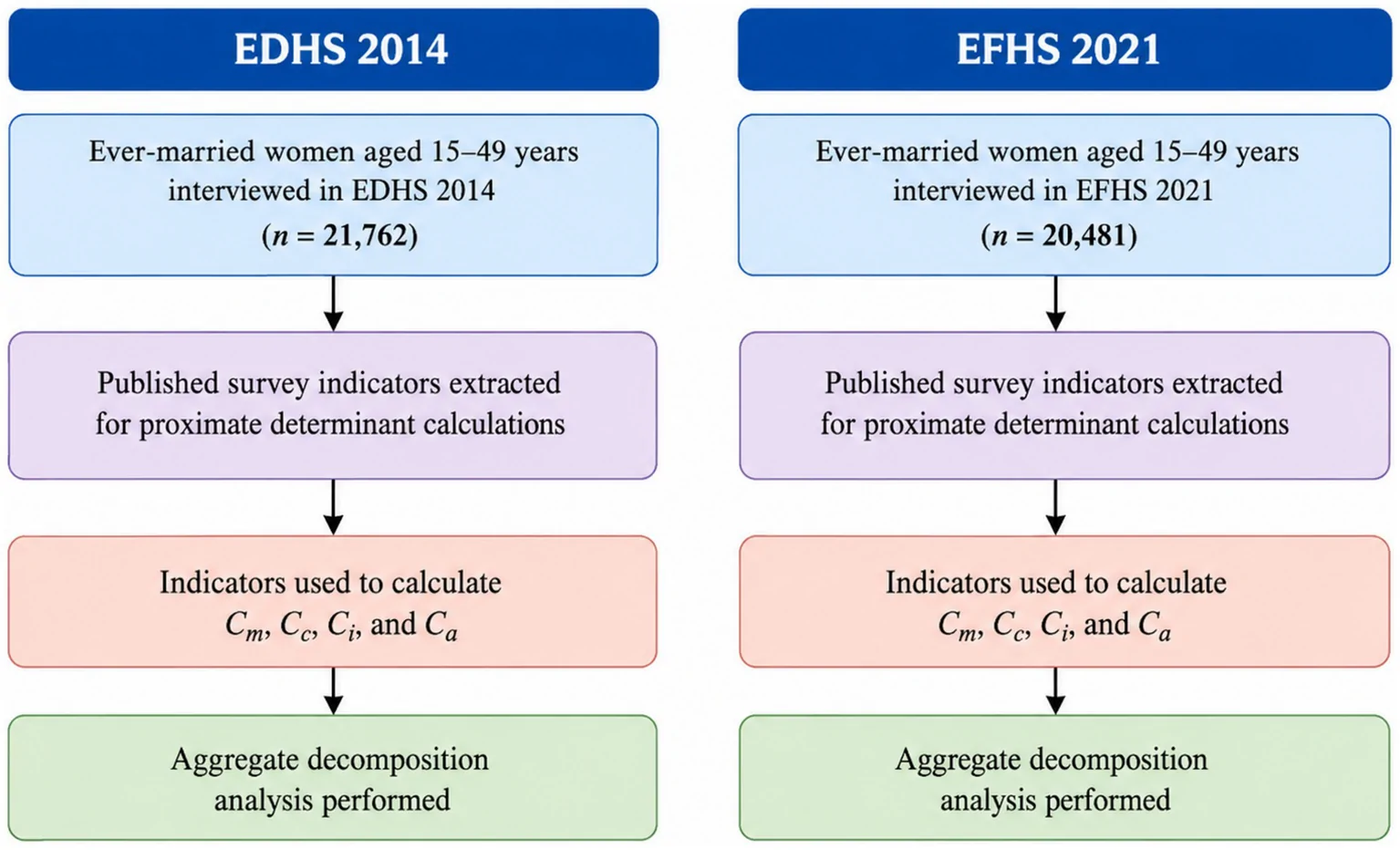
Data-source and indicator-extraction process for the EDHS 2014 and EFHS 2021 proximate determinants analysis. No individual-level exclusions or imputations were performed; the analysis was based on aggregate indicators reported in the survey publications.

### 3.2 Applied model

#### 3.2.1 Model specification

This study applies the aggregate Bongaarts-Potter proximate determinants model (Bongaarts, 1978; Bongaarts and Potter, 1983):

TFR=Cm×Cc×Ci×Ca×TF

where *C_m_* is the marriage index, *C_c_* the contraception index, *Ca* the induced abortion index, *Cᵢ* the postpartum infecundability index, and TF denotes total fecundity rather than total fertility rate. TF represents the hypothetical fertility level in the absence of the modeled fertility-inhibiting effects, whereas TFR represents observed period fertility (Bongaarts, 1978; Bongaarts and Potter, 1983). The primary analysis uses the conventional TF value of 15.3 births per woman. Although Bongaarts (2015) subsequently proposed refinements to improve the model under contemporary reproductive conditions, the original aggregate framework was retained because the published Egyptian survey indicators do not provide all variables required for implementation of the revised age-specific model (see Table 2).

**Table 2:** Definitions of the proximate determinant’s indices used in the Bongaarts’ model.

Index	Symbol	Definition
Marriage	*Cm*	Index of marriage (equals one if all women of reproductive age are married and zero in the absence of marriage)
Contraception	*CC*	Index of contraception (equals one in the absence of contraception and zero if all fecund women use 100% effective contraceptive methods)
Postpartum infecundability	*Cᵢ*	Index of postpartum infecundability (equals one in the absence of lactation and postpartum abstinence and zero if the duration of infecundability is infinite)
Induced abortion	*Ca*	Index of induced abortion (equals one in the absence of induced abortion and zero if all pregnancies are aborted)
Total fecundity	*TF*	Total fecundity representing fertility in the absence of the effects of the four proximate determinants

#### 3.2.2 Calculation of proximate determinant indices

The marriage index was calculated as:

$$c_{m}=\frac{\sum [m(a)\times g(a)]\;}{\sum [g(a)]}=\frac{\mathrm{TFR}}{\mathrm{TM}}$$

where *m(a)* is the age-specific proportion currently married, *g(a)* is the age-specific marital fertility rate, and TM is total marital fertility (Bongaarts, 1978; Bongaarts and Potter, 1983).

The contraception index was calculated as:

Cc=1−(1.08×U×e)

where *U* represents the prevalence of contraception among married women currently, and e denotes the weighted average effectiveness of the mix of contraceptive methods:

$$e=\frac{\sum [U(m)\times e(m)]\;}{U}$$

The effectiveness estimates were based on Bongaarts and Potter (1983): pill = 0.90, IUD = 0.95, injection/sterilization = 1.00, and other methods = 0.70. The multiplier of 1.08 was kept in the standard aggregate formulation (Bongaarts and Potter, 1983; Bongaarts, 2015) in order to maintain comparability to the base model. Since it has not been separately validated for modern Egypt or impacts C𝚌, use of the multiplier is considered an assumption regarding methodology.

Reliable induced abortion data were unavailable; therefore:

$$C_{a}=1$$

This is a computational assumption and does not imply that induced abortion was absent or had no fertility-inhibiting effect. Any unmeasured abortion effect may contribute to discrepancies between modeled and observed fertility.

The postpartum infecundability index was estimated as:

$$C_{i}=\frac{20\;}{\left(18.5+i\right)}$$

where *i* is the average duration of postpartum infecundability in months (Bongaarts and Potter, 1983).

#### 3.2.3 Assumptions and limitations

For the purpose of this analysis, Ca was set equal to 1 because reliable induced abortion data were unavailable in the published EDHS 2014 and EFHS 2021 indicators. This is a computational assumption and should not be interpreted as evidence that induced abortion was absent or had no fertility-inhibiting effect in Egypt. Consequently, any unmeasured fertility-inhibiting effect of induced abortion may contribute to differences between model-estimated and observed fertility. No change in fecundability or sterility over this short period was assumed, consistent with standard practice in period decompositions. Differences in reference periods, measurement, omitted proximate determinants, or model assumptions may also contribute to discrepancies between model-estimated and observed TFRs (Bongaarts, 2015).

#### 3.2.4 Statistical approach

A descriptive methodology was applied which utilized deterministic computations based on the Bongaarts Proximate Determinant (PD) framework. Indicators of fertility and reproductive health data from surveys were used to calculate the PD index values to break down the total changes in fertility during 2014–21. Due to an excessive interaction component produced by the preliminary arithmetic decomposition, the relative influence of each proximate determinant was analyzed via logarithmic decomposition of the multiplicative model. The contribution of each proximate determinant was assessed from its logarithmic change relative to the combined logarithmic change in the modeled determinants. This approach avoids a separate arithmetic interaction component. Because the model-estimated TFRs differ from the observed national TFRs, these contributions are interpreted as components of the model-implied fertility change rather than as exact causal shares of the observed fertility decline.

The standard error of estimates and confidence intervals were not determined, since this research effort emphasized descriptive decomposition of fertility change as opposed to statistical hypothesis testing. A regression analysis did not occur since the primary objective was to determine the net magnitude of aggregate fertility change over time, as opposed to quantifying relationships, determining predictors, or estimating causality at an individual level. Therefore, changes observed between survey years were described as such, but not statistically compared. Accordingly, the results are interpreted as descriptive associations and model-based contributions rather than causal effects.

#### 3.2.5 Sensitivity analysis

To evaluate the sensitivity of the model-estimated fertility levels to the total fecundity assumption, the Bongaarts model was recalculated using TF = 17 births per woman, as discussed by Horne (1993), in addition to the primary TF = 15.3 specification (Bongaarts and Potter, 1983). The resulting model-estimated TFRs were compared with the observed TFRs for 2014 and 2021. Because the same TF value was applied to both years within each specification, this analysis evaluates the sensitivity of model fit to the assumed total fecundity level rather than treating TF as a changing proximate determinant.

## 4 Results

### 4.1 Characteristics of the surveys and study populations

Ever-married women aged 15–49 years were included in both surveys to ensure comparison of the same demographic population from which the fertility and contraceptive indicators were derived. Aggregate fertility and reproductive-behavior indicators were analyzed because they provide the measures required to calculate the Bongaarts proximate determinant indices and decompose changes in fertility (see Table 3).

**Table 3:** Background characteristics of ever-married women included in EDHS 2014 and EFHS 2021.

Characteristic	EDHS 2014, weighted, *n* (%)	EFHS 2021, weighted, *n* (%)
Place of residence		
Urban	7,623 (35.0)	7,797 (38.1)
Rural	14,139 (65.0)	12,684 (61.9)
Educational attainment		
No education	5,232 (24.0)	3,362 (16.4)
Some primary education	1,334 (6.1)	1,025 (5.0)
Primary completed/some secondary	3,796 (17.4)	4,258 (20.8)
Secondary completed/higher education	11,400 (52.4)	11,837 (57.8)
Marital status		
Currently married	20,460 (94.0)	19,044 (93.0)
Formerly married	1,302 (6.0)	1,437 (7.0)
Total ever-married women	21,762 (100.0)	20,481 (100.0)

Values are weighted numbers and percentages. Age-specific characteristics are presented separately in Table 4 because they are used directly to calculate the marriage index. Minor differences in totals may occur because of rounding. Source: Ministry of Health and Population [Egypt] et al. (2015) and Central Agency for Public Mobilization and Statistics (CAPMAS), (2022).

### 4.2 Proximate determinant indices and fertility decline during 2014–2021

#### 4.2.1 Index of marriage (*C_m_*)

The marriage index Cm was derived from the age-specific percentages of women who are presently married along with their associated age-specific marital fertility rates. It is distinct from the straightforward ratio of married women of reproductive age since the impact of marriage on fertility differs among age categories.

The marriage index was determined by utilizing the subsequent formula:

$$C_{m}=\frac{\sum m(a)g(a)}{\sum g(a)}=\frac{\mathrm{TFR}}{\mathrm{TM}}$$

Where: *m_(a)_* = age-specific proportion of females currently married; *g_(a)_* = Age-specific marital fertility rate; *TFR* = total fertility rate; *TM* = total marital fertility rate.

Table 4 shows an increase in percent of women currently married as a function of increasing age, reaching its peak for those aged 35–39 years old for each year surveyed. A decrease in the marriage index (*C_m_*), dropping from 0.56 in 2014 to 0.39 in 2021, indicates stronger fertility inhibition associated with reduced marriage exposure.

**Table 4:** Proportion of currently married women by age, age-specific marital fertility rate, and estimated index of marriage (C_m_) in Egypt, 2014–2021.

Years	2014			2021		
Age group	m(a)	g(a)	m(a) × g(a)	m(a)	g(a)	m(a) × g(a)
15–19	0.144	389	56	0.073	685	50
20–24	0.596	357	213	0.467	364	170
25–29	0.845	237	200	0.798	212	169
30–34	0.899	149	134	0.889	126	112
35–39	0.91	76	69	0.897	59	53
40–44	0.883	19	17	0.876	15	13
45–49	0.83	5	4	0.836	2	2
Total		1,232	693		1,463	569
Index (C_m_)	C_m_ = 0.56			C_m_ = 0.39		

Source: Calculated from the Egypt Demographic and Health Survey (Ministry of Health and Population [Egypt] et al., 2015) and Egyptian Family Health Survey (Central Agency for Public Mobilization and Statistics (CAPMAS), 2022).

The index of marriage ( Cm ) is calculated as:

$$c_{m}=\frac{\sum [m(a)\times g(a)]\;}{\sum [g(a)]}$$

Using the data from Table 4:

For 2014:

$$c_{m}=\frac{693}{1232}=0.562$$

For 2021:

$$c_{m}=\frac{569}{1463}=0.389$$

Marriage Index fell from 0.56 in 2014 to 0.39 by 2021. Therefore, it is possible that in 2021 marriage was inhibiting fertility more strongly than in 2014. It has been reported that in other Arab countries changes in what people expect from marriage, as well as the increasing effects of economic pressure and beliefs regarding marital instability are having an increasing impact on when people get married and how they form their families (Rehim et al., 2020).

This decrease in *Cm* agrees with a reduction of marriage exposure at younger reproductive ages; for instance, the percentage of currently married among women aged 20–24 decreased from 59.6 percent in 2014 to 46.7% in 2021 (Table 4). However, the aggregated data do not permit this change to be attributed exclusively to later age at first marriage.

#### 4.2.2 Index of contraception (*C_c_*)

The contraception index ( Cc ) evaluates the fertility-reducing impact of contraceptive use by assessing both the prevalence of contraceptive use and the effectiveness of the combination of contraceptive methods. The index was determined using this equation:

Cc=(1−(1.08×U×e))

Where: *C_c_* = Index of contraception; *U* = proportion of currently married women using contraception; *e* = weighted average effectiveness of the contraceptive method mixes.

The weighted average effectiveness of contraception was estimated as

$$e=\frac{\sum (\mathrm{Um}\times \mathrm{em})}{U\;}$$

Where: Um = prevalence of use of each contraceptive method; em = effectiveness value assigned to each contraceptive method based on Bongaarts and Potter (1983)

The effectiveness values applied were: pill = 0.90, IUD = 0.95, injection/sterilization = 1.00, and other methods = 0.70.

Using the values presented in Table 5, the weighted average effectiveness and contraceptive index were calculated separately for each survey year. For 2014:

$$e_{2014}=\frac{\left(0.160\times 0.90)+(0.301\times 0.95)+(0.097\times 1.00)+(0.022\times 0.70\right)}{0.58}=0.935$$

Cc2014=1−(1.08×0.58×0.935)=0.414≈0.41

**Table 5:** Estimation of the index of contraception (C𝚌) in Egypt, 2014–2021.

Contraceptive method	2014 (Um)	Effectiveness (em)	2021 (Um)	Effectiveness (em)
Pill	0.160	0.90	0.195	0.90
IUD	0.301	0.95	0.294	0.95
Injection and sterilization	0.097	1.00	0.122	1.00
Other methods	0.022	0.70	0.044	0.70
Contraceptive prevalence (U)	0.58	—	0.66	—
Weighted average effectiveness (e)	0.935	—	0.921	—
Contraceptive index (Cc)	0.41	—	0.34	—

The contraceptive index (Cc) was calculated using the Bongaarts and Potter (1983) formula: Cc = 1 − (1.08 × *U* × e), where *U* represents contraceptive prevalence and e represents the weighted average effectiveness of the contraceptive method mix. The weighted average effectiveness was calculated as e = Σ (*U*m​ × em)/*U*. Method-specific prevalence values may not sum exactly to total contraceptive prevalence because of rounding. Source: Calculated from the Egypt Demographic and Health Survey (Ministry of Health and Population [Egypt] et al., 2015) and Egyptian Family Health Survey (Central Agency for Public Mobilization and Statistics (CAPMAS), 2022).

For 2021:

$$e_{2021}=\frac{\left(0.195\times 0.90)+(0.294\times 0.95)+(0.122\times 1.00)+(0.044\times 0.70\right)}{0.66}=0.921$$

Cc2021=1−(1.08×0.66×0.921)=0.344≈0.34

In 2014, the user-weighted average effectiveness of the contraceptive method mix had been 0.935 and by 2021 it had dropped to 0.921; the contraceptive index (C_c_) likewise decreased from around 0.41 to 0.34. Table 5 provided the data upon which the distribution of the most frequently used contraceptives was calculated as well. The percentage of contraceptive users who were using an intrauterine device (IUD), pills, injections/sterilizations, and other types of contraceptives in 2014 was 51.9, 27.6, 16.7 and 3.8%, respectively. In contrast, by 2021 the percentages were 44.5, 29.5, 18.5 and 6.7%, respectively.

Although the IUD was still the predominant type of contraceptive in each year’s survey, its share among contraceptive users decreased by 7.4 percentage points, from 51.9% in 2014 to 44.5% in 2021, while the percentage of users of pills, injections/sterilizations, and other types of contraception rose. However, since there was an increase in the total percentage of women using contraceptives from 58 to 66%, the slight decrease in the weighted average effectiveness of the method mix was offset by this larger increase.

#### 4.2.3 Index of induced abortion (c_a_)

Statistics related to induced abortion are not gathered in the Egypt Demographic and Health Survey (EDHS) and the Egyptian Family Health Survey (EFHS), as induced abortion is legally restricted in Egypt. Because reliable indicators required to estimate the abortion index were unavailable, *Ca* was set equal to 1 for both survey periods as a computational assumption. This value should not be interpreted as evidence that induced abortion was absent or exerted no fertility-inhibiting effect.

#### 4.2.4 Index of postpartum Infecundability (c_i_)

Postpartum infecundability is a mechanism that reduces fertility in women by delaying ovulation and the return of menstrual cycling after childbirth. Among women not using contraception, prolonged breastfeeding may extend postpartum amenorrhea, delay the return of ovulation, and decrease exposure to conception. The postpartum infecundability index was estimated by Bongaarts and Potter (1983) with the following formula:

$$\mathrm{Ci}=\frac{20}{(18.5+i)\;}$$

where *i* is the average duration of postpartum infecundability caused by breastfeeding or postpartum abstinence.

Table 6 shows that the mean duration of postpartum amenorrhea increased from 6.8 months in 2014 to 7.4 months in 2021. Because a comparable measure of postpartum abstinence was unavailable in the published survey indicators, postpartum amenorrhea was used as the available proxy for the duration of postpartum infecundability (i) in calculating *C_i_*. Consequently, the index of postpartum infecundability (*C_i_*) decreased from 0.79 in 2014 to 0.77 in 2021, indicating a modest increase in fertility inhibition from postpartum infecundability over the study period.

**Table 6:** Assessment of the average duration of postpartum infecundability in Egypt, 2014–2021.

Years	Mean Duration of breastfeeding in Months	Postpartum Amenorrhea in Months	C_i_
2014	17.7	6.8	0.79
2021	17.8	7.4	0.77

Source: Calculated from the Egypt Demographic and Health Survey (Ministry of Health and Population [Egypt] et al., 2015) and Egyptian Family Health Survey (Central Agency for Public Mobilization and Statistics (CAPMAS), 2022).

Because a comparable published measure of postpartum abstinence was unavailable for both survey years, postpartum amenorrhea was used as the available proxy for the duration of postpartum infecundability (i) in calculating C_i_.

#### 4.2.5 Model-estimated TFR and sensitivity to Total fecundity

Table 7 compares the observed TFR with the fertility level reproduced by the Bongaarts model. The calculations were rechecked using the unrounded values of the proximate-determinant indices derived in Tables 4–6. With TF = 15.3 births per woman, the model-estimated TFR was 2.82 births per woman in 2014 and 1.58 in 2021, compared with observed TFRs of 3.50 and 2.85, respectively. The corresponding model–observation differences were 0.68 births in 2014 and 1.27 births in 2021.

**Table 7:** Model-estimated and observed total fertility rates and sensitivity to the total fecundity assumption in Egypt, 2014–2021.

Year	Marriage index (C_m_)	Contraceptive index (C_c_)	Postpartum index (C_i_)	Abortion index (C_a_)	Model TFR (TF = 15.3)	Observed TFR	Difference	Model TFR (TF = 17)	Difference
2014	0.56	0.41	0.79	1.00	2.82	3.50	0.68	3.13	0.37
2021	0.39	0.34	0.77	1.00	1.58	2.85	1.27	1.76	1.09

Source: Authors’ calculations based on aggregate indicators reported in the EDHS 2014 and EFHS 2021 (Ministry of Health and Population [Egypt] et al., 2015; Central Agency for Public Mobilization and Statistics (CAPMAS), 2022).

The model-estimated TFR was calculated as TFR = Cm × Cc × Ci × Ca × TF. Calculations were performed using the unrounded proximate-determinant index values underlying the rounded values displayed in the table. TF = 15.3 represents the primary specification, whereas TF = 17 represents the sensitivity analysis. Difference = observed TFR−model-estimated TFR. The remaining discrepancies indicate that the measured proximate determinants and assumed total fecundity values do not fully reproduce the observed fertility levels, particularly in 2021.

Thus, although the measured indices moved in a fertility-inhibiting direction, the four-index model did not reproduce the observed fertility levels closely, particularly in 2021. The model implied a decline of approximately 1.24 births per woman between the two survey years, whereas the observed decline was 0.65 births per woman. This discrepancy indicates that the magnitude of change generated by the specified aggregate model should not be interpreted as an exact explanation of the observed national fertility decline.

As a sensitivity analysis, TF was increased from 15.3 to 17 births per woman. The resulting model-estimated TFRs increased to 3.13 in 2014 and 1.76 in 2021. Although TF = 17 reduced the absolute difference between modeled and observed fertility, particularly in 2014, a substantial discrepancy remained in 2021. Moreover, because the same TF value was applied to both survey years, changing TF altered the level of the modeled TFR but not the relative pattern of change generated by the proximate-determinant indices.

#### 4.2.6 Decomposition of the indices and their contributions in fertility decline

Change in fertility levels from one time period to another is due to change(s) in one or more Proximal Determinants. Because the preliminary arithmetic decomposition produced a large interaction component, the revised analysis used logarithmic decomposition to assess the relative contribution of each measured proximate determinant to the fertility change reproduced by the Bongaarts model (see Tables 8).

**Table 8:** Logarithmic decomposition of the model-implied fertility change in Egypt, 2014–2021.

Factor	2014 index	2021 index	Logarithmic change	Relative contribution to model-implied change (%)
Marriage	0.562500	0.388927	−0.3690	63.7
Contraceptive practice	0.414262	0.343792	−0.1865	32.2
Postpartum infecundability	0.790514	0.772201	−0.0234	4.1
Induced abortion	1.000000	1.000000	0.0000	0.0
Combined product of indices	0.184207	0.103251	−0.5789	100.0

Authors’ calculations based on the proximate-determinant indices derived from the EDHS 2014 and EFHS 2021. Calculations were performed using the unrounded proximate-determinant index values underlying the rounded values reported elsewhere in the manuscript. Relative contributions were calculated by dividing each determinant’s logarithmic change by the combined logarithmic change. These percentages represent the relative composition of the model-implied fertility change, not shares of the observed national TFR decline. The induced abortion contribution is 0.0% because Ca​ was fixed at 1 in both survey years as a computational assumption.

The logarithmic change associated with each proximate determinant was calculated as follows.

For the marriage index:

$$\ln(\frac{C_{m,2021}}{C_{m,2014}})=\ln(\frac{0.388927}{0.562500})=-0.3690$$

For the contraception index:

$$\ln(\frac{C_{c,2021}}{C_{c,2014}})=\ln(\frac{0.343792}{0.414262})=-0.1865$$

For the induced abortion index:

$$\ln(\frac{C_{a,2021}}{C_{a,2014}})=\ln(\frac{1.000}{1.000})=0.0000$$

For the postpartum infecundability index:

$$\ln(\frac{C_{i,2021}}{C_{i,2014}})=\ln(\frac{0.772201}{0.790514})=-0.0234$$

The combined logarithmic change in the measured proximate determinants was:

−0.3690+(−0.1865)+0.0000+(−0.0234)=−0.5789

The contribution of each proximate determinant was determined by dividing its logarithmic variation by the cumulative logarithmic variation.

For marriage:

$$\frac{-0.3690}{-0.5789}\times 100=63.7\%$$

For contraception:

$$\frac{-0.1865}{-0.5789}\times 100=32.2\%$$

For postpartum infecundability:

$$\frac{-0.0234}{-0.5789}\times 100=4.1\%$$

For induced abortion:

$$\frac{0.0000}{-0.5789}\times 100=0.0\%$$

The decomposition using logarithms indicates that the largest portion (approximately 63.7%) of the decline in modeled fertility due to reduced marriage exposure was caused by the decrease in marriage exposure. The second largest portion of the total fertility decline was changes in contraceptive use (approximately 32.2%), while postpartum infecundability accounted for approximately 4.1%. The induced abortion element was 0.0% since *Ca* was set at 1 in both survey years as a computational assumption, not because it was shown that induced abortion has no effect on fertility.

These percentages represent the comparative makeup of the fertility change generated by the Bongaarts model and should not be seen as precise portions of the actual national fertility reduction. The model-estimated TFR fell from about 2.82 births per woman in 2014 to 1.58 in 2021, reflecting a reduction of around 43.9%, while the observed TFR dropped from 3.50 to 2.85 births per woman, indicating a decline of about 18.6%. The significant difference in the extent of change suggests that the measured proximate determinants and assumptions of the aggregate model do not completely explain Egypt’s actual fertility path. Thus, the decomposition outcomes are employed to demonstrate the relative impact of each assessed factor on the model-derived fertility change instead of its causal portion of the noted fertility reduction.

## 5 Discussion

### 5.1 Interpretation of key findings

The data suggest that Egypt has experienced an overall decline in total fertility rate between 2014 and 2021 that was associated with changes in several proximate determinants. As the marriage index declined from 0.56 to 0.39 exposure to marital fertility for women was reduced. There was an increase in the inhibitory effects of contraception on fertility which was indicated by the decline in the contraceptive index from 0.41 to 0.34. Although there was a slight reduction in the postpartum infecundability index from 0.79 to 0.77, this indicates a small increase in the amount of time it takes after delivery for a woman to be fertile again. The logarithmic decomposition shows that marriage accounted for roughly 63.7% of the fertility variation generated by the model, contraception 32.2%, and postpartum infecundability 4.1%. These percentages depict the relative makeup of the model-derived change rather than the causal contributions to the observed national decline in fertility.

The more pronounced contraceptive inhibiting effect was seen despite a very small decrease from 0.935 to 0.921 in the overall weighted mix of methods used due to an increase in the percentage of the population using contraception from 58 to 66%. A significant reduction in marriage exposure for reproductive-aged females has been noted as well; there is evidence that the percent of females currently married among females 20–24 years old decreased from 59.6 to 46.7%.

A significant limitation is that the model did not accurately replicate observed fertility. With a TF of 15.3, the model-estimated TFR decreased from about 2.82 in 2014 to 1.58 in 2021, while the observed decrease was from 3.50 to 2.85. The assessed proximate determinants thus offer an organized explanation of the relative behavioral factors linked to fertility change but do not deliver a full quantitative rationale for the noted national decline.

### 5.2 Evaluation against earlier Egyptian research

The importance of marriage and contraception in the reduction of fertility rates in Egypt has changed over the years. Zaky (1999), examining the years 1980–1995, concluded that contraception played a crucial role in decreasing fertility. At that time, contraceptive prevalence was lower and method mix was different, allowing for greater fertility-inhibiting effects from increased coverage.

During the second half of the 1990s, Eltigani (2003) determined that the fertility decline had slowed down. He found that it was the reproductive behaviors of women in households with higher to medium standards of living that contributed most to the slowing of this trend. Eltigani (2009) reported that the difference in the age at marriage for women represented one of the primary factors contributing to differences in rates of decline in fertility in Egypt compared to Tunisia. In contrast to Zaky’s earlier findings, marriage represents the largest component of the model-implied change in 2014–2021. This difference is consistent with the studies examining different stages of Egypt’s fertility transition: contraceptive prevalence was already relatively high by 2014, while marriage exposure declined markedly thereafter. The findings are also broadly consistent with Eltigani’s emphasis on marriage timing, although the present aggregate analysis cannot establish the socioeconomic causes underlying changes in marriage behavior.

### 5.3 Comparison with cross-National Evidence

The cross-national applications summarized in Table 1 show considerable variation in which proximate determinant dominates fertility decline. In Ethiopia, marriage was the main barrier in 2011, while postpartum infecundability was prevalent in 2016 (Laelago et al., 2019). In Eswatini, contraception was the primary factor (Chemhaka and Odimegwu, 2019). In Namibia (Palamuleni, 2017) and Timor-Leste (Hogan et al., 2025), marriage was the main factor, while in Mongolia (Spoorenberg, 2009), contraception was more prevalent.

In summary, Egypt’s results support that the proximate determinants may be variable depending on the specific circumstances. Each circumstance will have its own set of variables for determining what will influence a woman’s likelihood to use a modern family planning contraceptive. Therefore, this study does not conflict with previous studies in which postpartum infecundability or contraception were found to be the primary influences of FP contraceptive usage among women.

### 5.4 Theoretical implications

The results highlight the necessity for differentiating between the proportional contribution of the proximal causes contained in the Bongaarts model and its ability to replicate the observed fertility. Although marriage was the single largest modeled component (followed by contraception and postpartum infecundability), the large difference between the model’s output and the actual fertility rate demonstrates that this model cannot completely account for Egypt’s fertility transition. Thus, it should be interpreted in this study as an organized decomposition of measurable proximal mechanisms, instead of as a total explanatory mechanism.

Second, the endogeneity of fertility preferences highlighted in section 2.1 is relevant from an empirical perspective. Bongaarts (2024) and N-IUSSP (2024) indicate that contraceptive behavior may respond to reproductive preferences rather than operate independently of them. Thus, Egypt’s increased contraceptive prevalence is compatible with, but does not demonstrate, changes in desired family size. Because fertility preferences were not measured directly in this aggregate analysis, they remain a possible background pathway rather than an explanation established by the study.

### 5.5 Methodological reflections

The model applies the standard total fecundity value of 15.3 proposed by Bongaarts and Potter (1983), supporting comparison with earlier studies. Horne (1993), however, proposed a value of 17 for Arab countries, including Egypt, to prevent negative decomposition percentages. Sensitivity analysis using TF = 17 increased the model-estimated TFR from 2.82 to approximately 3.13 in 2014 and from 1.58 to approximately 1.76 in 2021. The alternative assumption reduced the model–observation discrepancy, particularly in 2014, but did not eliminate it in 2021. Because TF was held constant across years within each specification, changing TF affected modeled fertility levels but not the relative contributions of the proximate determinants.

The computational assumption Ca​ = 1, necessitated by the absence of reliable induced abortion indicators, also requires caution. This assumption should not be interpreted as evidence that induced abortion was absent or had no fertility-inhibiting effect.

The remaining model–observation discrepancy may reflect differences in reference periods, omitted or imperfectly measured proximate determinants, and fixed model parameters. It’s greater magnitude in 2021 warrants caution in interpreting the decomposition as an explanation of the observed 0.65-birth decline. Future research using respondent-level microdata could improve temporal alignment and evaluate revised model specifications.

### 5.6 Study contributions

This paper provides a new proximate determinants analysis to explain Egypt’s post-stall fertility decline through the use of data from two rounds of surveys (EDHS 2014 and EFHS 2021). The findings indicate that contraceptive prevalence and the effectiveness of methods should be evaluated together when analyzing the fertility-reducing impact of contraception. In addition, the study demonstrates that reduced marriage exposure and increased contraceptive inhibition were the key measurable mechanisms for this decline. Additionally, it is important to understand which portions of the decline can be accounted for by the Bongaarts framework and what limitations there may have been in applying it to the case of Egypt.

## 6 Conclusion and recommendations

### 6.1 Summary of findings

This study applied Bongaart’s proximate determinants framework to analyze fertility change in Egypt using data collected from the EDHS (2014) and EFHS (2021). Overall Fertility Rate has been declined as it went down from 3.50 births/woman in 2014 to 2.85 births/woman by 2021. Marriage Index declined as well from 0.56 to 0.39. Decline in Marriage Exposure is consistent with the declining exposure that younger women are having to marital fertility. The contraceptive index dropped from 0.41 to 0.34 showing contraception had a larger inhibitory effect on fertility in 2021. Although there was a slight drop in the overall effectiveness of the contraceptive method mix, the increase in contraceptive use from 58 to 66% was greater than this decrease.

The logarithmic decomposition indicated that marriage represented approximately 63.7% of the fertility change accounted for in the estimated models as well as contraception at 32.2%, and postpartum at 4.1%. Thus, these values indicate what proportion of the total estimated model fertility change can be attributed to each determinant; they represent the proportions of the estimated model implied fertility changes rather than share of cause of the observed declines. The estimated total Fertility Rate (TFR) declined from an estimated 2.82 to an estimated 1.58 while the actual total Fertility Rate (TFR) decreased from 3.50 to 2.85; therefore, it is clear that the measured proximate causes do not capture all of Egypt’s total fertility rate decrease.

### 6.2 Policy implications

The study results indicate that Family Planning Policy will need to address issues such as contraceptive use, availability of contraception, choice regarding the best method for each woman, quality of service and continued use of selected methods as interrelated objectives. While there is an improvement in contraceptive prevalence from 58 to 66% as well as a small decrease in weighted method-mix effectiveness from 0.935 to 0.921, this supports the emphasis on voluntary access to a wide variety of effective methods, appropriate counseling, management of side effects, and continuity of care rather than contraceptive prevalence alone.

The substantial reduction in marriage exposure, including the decline in the proportion currently married among women aged 20–24 from 59.6 to 46.7%, also highlights the demographic importance of marriage patterns. However, because the aggregate analysis cannot establish why marriage exposure changed, the findings do not justify attributing this change to a specific policy or socioeconomic factor. Educational and employment opportunities that support voluntary family-formation decisions may complement broader population and development objectives (Rehim et al., 2023).

Finally, improving equitable access to reproductive-health services, including outreach and mobile services for women wishing to space or limit births, remains relevant to documented unmet family-planning needs (Ahram Online, 2022), although the effectiveness of such interventions was not directly evaluated in this study.

### 6.3 Strengths of the study

This study has a number of advantages. The study uses two large-scale nationwide sample surveys—the EDHS in 2014 and the EFHS in 2021 that have wide population coverage and enable comparisons over time. In addition, the study applies an existing Bongaarts framework of proximate causes (behavioral mechanisms) for measuring the pathways behind fertility changes. Based on current understanding, this is the inaugural study to apply EFHS 2021 data within the Bongaarts proximate determinants model to analyze Egypt’s fertility decrease following the 2014 pause. The revised analysis further strengthens methodological transparency by distinguishing model-implied contributions from the observed fertility decline and by evaluating the sensitivity of model estimates to an alternative TF value.

### 6.4 Limitations of the study

This study is limited. In this respect, it can show associations for how proximate determinants of fertility influence or contribute to fertility decline, but the Bongaarts Framework does not demonstrate why certain variables have changed. Because the analysis is descriptive and deterministic, the estimated contributions should not be interpreted as causal effects.

Second, Ca = 1 was imposed because reliable induced abortion indicators were unavailable; this computational assumption does not demonstrate that induced abortion was absent or had no fertility-inhibiting effect (Central Agency for Public Mobilization and Statistics (CAPMAS), 2022; Ministry of Health and Population [Egypt] et al., 2015).

Third, the primary analysis assumed TF = 15.3. Sensitivity analysis using TF = 17 reduced the discrepancy between modeled and observed fertility but did not eliminate it, particularly in 2021 (Bongaarts, 2015; Horne, 1993).

Fourth, the national aggregate analysis may mask regional variations in marriage patterns, contraceptive use, education, and socioeconomic conditions. The reliance on published aggregate indicators rather than respondent-level microdata also limits adjustment for differences in reference periods and measurement and may contribute to the discrepancy between model-estimated and observed TFRs. The seven-year research duration might also miss slow shifts in reproductive behavior.

Ultimately, intentions regarding fertility can affect marital decisions and the use of contraception, suggesting that the immediate factors could be linked to fertility desires instead of functioning separately (N-IUSSP, 2024; Bongaarts, 2024).

## Data Availability

Publicly available datasets were analyzed in this study. This data can be found here: The 2014 Egypt Demographic and Health Survey data are publicly available from The DHS Program (https://dhsprogram.com) upon request and approval. The 2021 Egyptian Family Health Survey data are available from Egypt’s Central Agency for Public Mobilization and Statistics (CAPMAS) upon request. The authors confirm that they had no special access privileges to these data.
